# A Comparison between Two Instruments for Assessing Dependency in Daily Activities: Agreement of the Northwick Park Dependency Score with the Functional Independence Measure

**DOI:** 10.1155/2012/769513

**Published:** 2012-11-18

**Authors:** Siv Svensson, Katharina Stibrant Sunnerhagen

**Affiliations:** Institute of Neuroscience and Physiology/Rehabilitation Medicine, Sahlgrenska Academy, University of Gothenburg, 3rd floor, Per Dubbsgatan 14, 413 45 Gothenburg, Sweden

## Abstract

*Background*. There is a need for tools to assess dependency among persons with severe impairments. *Objectives*. The aim was to compare the Functional Independence Measure (FIM) and the Northwick Park Dependency Score (NPDS), in a sample from in-patient rehabilitation. *Material and Methods*. Data from 115 persons (20 to 65 years of age) with neurological impairments was gathered. Analyses were made of sensitivity, specificity, positive predictive value, and negative predictive value. Agreement of the scales was assessed with kappa and concordance with Goodman-Kruskal's gamma. Scale structures were explored using the Rank-Transformable Pattern of Agreement (RTPA). Content validation was performed. *Results*. The sensitivity of the NPDS as compared to FIM varied between 0.53 (feeding) and 1.0 (mobility) and specificity between 0.64 (mobility) and 1.0 (bladder). The positive predictive value varied from 0.62 (mobility) to 1.0 (bladder), and the negative predictive value varied from 0.48 (bowel) to 1.0 (mobility). Agreement between the scales was moderate to good (four items) and excellent (three items). Concordance was good, with a gamma of −.856, an asymptotic error (ase) of .025, and *P* < .000. The parallel reliability between the FIM and the NPDS showed a tendency for NPDS to be more sensitive (having more categories) when dependency is high. *Conclusion*. FIM and NPDS complement each other. NPDS can be used as a measure for severely injured patients who are sensitive when there is a high need of nursing time.

## 1. Introduction

There is a need for tools to assess dependency among persons with severe impairments. It seems that the number of persons with dependency as a result of acquired brain injuries is increasing or at least more are referred for rehabilitation. This includes not only traditional active rehabilitation aiming at discharge to the home but also for specific treatment of spasticity in persons who are totally dependent. Outcome assessment tools have not only to be valid but also responsive enough to detect changes during rehabilitation [1]. This is a matter of quality of care, for the individual and for the payer.

There are different instruments that aim to describe activities of daily living (ADL) and levels of dependency/independency. Both the Barthel ADL index [2] and the FIM (Functional Independence Measure) [3, 4] can be considered “golden standards” for ADL assessment and both have known floor and ceiling effects. When impairments are severe, the improvements after an intervention are sometimes small, and the feeling of the staff is that achievements are not reflected in the traditional ADL instruments.

The Northwick Park Dependency Score (NPDS) was developed to meet this need [5]. The goal of the NPDS was to assess the nursing time required in the rehabilitation, providing another way to assess dependency and perhaps better reflecting small changes in dependency. Its reliability and validity have been studied [6]. The instrument has been translated into Swedish [7]. Although a very recent review was published [8] that covered five studies, only one study included more than 100 patients [6]. We thus believe that there is a need for further validation studies of the instrument. One way to perform a content validation of an instrument is by linking to the International Classification of Functioning (ICF).

The aim of this study was to compare the assessments of ADL dependency made with two different scales, the FIM and the NPDS, in a sample of persons receiving in-patient rehabilitation. A second aim was to perform a content validation of the scales by using the ICF.

## 2. Subjects and Methods

Data were gathered from 115 persons, 47 women and 68 men, with a mean age of 50 years (ranging from 20–65 years). Acquired brain injury was the most common cause of the need of in-patient rehabilitation (77% stroke, the most common, 64 persons, and 22 traumatic brain injury). The remaining patients had other neurological diagnoses (multiple sclerosis, Guillain-Barre, or spinal cord injury) requiring rehabilitation.

### 2.1. Instruments

The FIM instrument [3, 4] was designed to measure the degree of disability experienced, changes over time, and the effectiveness of rehabilitation. It intends to measure severity defined in terms of the need of assistance. The FIM can be used with any rehabilitation client. There is a manual, and the use of FIM requires training. It is designed to be applied in people seven years of age and older. Each item is rated on a seven-point scale, from total assistance to complete independence (13 physical items and five cognitive/social items). Total scores range from 18 to 126, with 126 indicating independence.

NPDS [1] is designed to be used for assessments of the requirement of nursing time in a rehabilitation setting in order to evaluate the full range of dependency. According to the constructer, it seems to be particularly sensitive to small changes in dependency that would not be detected by other instruments. NPDS is divided into two sections: basic care needs (BCNs) and special nursing needs (SNN). A score of 100 indicates dependence in all items (BCH = 65, SNN = 35), and a score of 0 indicates independence in all items. Lower scores thus indicate that the person is more independent, where low dependence is <10, medium dependence is 10–25, and high dependence is >25. NPDS assessments are made by observation. No formal training is said to be needed to use the instrument. A manual is available.

### 2.2. Data Collection

The raters were trained in the use of FIM and had long experience of working in rehabilitation of neurologically impaired patients. The NPDS does not require formal training; the instrument is meant to be self-explanatory (it does, however, have a manual). This was given to the raters together with a scoring sheet. The FIM assessment was made first (immediately prior to discharge) and this was followed by the NPDS (BCN + SNN sections). The raters also collected descriptive information concerning the patient's age, sex, and diagnoses.

### 2.3. Comparison of Items of the FIM and NPDS

The procedure for comparison of the FIM and NPDS with the ICF was accomplished by two raters who were experienced in the field of clinical neurorehabilitation and familiar with the ICF. The comparison was carried out independently. The process and the final evaluation of appropriate codes were based on the independent ratings and subsequent discussions between the raters to reach consensus.

The study was approved by the Ethics Committee of the University of Gothenburg. All patients or their next of kin (if the patient could not read or understand) were given a letter containing information about the study. All gave their informed consent to participate.

### 2.4. Statistical Analysis

The data were analysed using the Statistical Program for the Social Sciences (SPSS) 16.0. Cross-tabulations were made to explore the precision of the instrument in identifying independency/dependency in personal ADL, where the items from the instruments were merged to resemble one another. The items from FIM served as the “golden standard.” Sensitivity, specificity, positive predictive values, and negative predictive values are presented. The alpha value was set to ≤0.05.

The agreement on dependency was assessed for the different ADL areas based on Cohen's unweighted kappa as a measure of agreement, *P* values, and values of percentage agreement (PA). Kappa coefficients between 0.40 and 0.80 are considered moderate to good, and those exceeding 0.80 are very good, while values below 0.40 are fair to poor.

Goodman-Kruskal's gamma was calculated to assess concurrent validity between the total score in the FIM and the total score in the BCN section of NPDS. Concordance is defined as a measure of the interchangeability of two scales, which means that, if two scales are concordant, they will produce the same ordering of individuals. Gamma is a measure based on the difference between the numbers of concordant and discordant pairs adjusted for ties on the marginal distribution. Gamma can vary between −1 and 1, where a value of 1 indicates perfect concordance, and the value of 0 indicates a total lack of concordance. If one scale is the reverse of the other, the value of −1 indicates total concordance [9]. The asymptotic standard error (ase) is given in the analysis as a measure of precision for gamma.

The rank-transformable pattern of agreement (RTPA) was used for parallel reliability between the FIM and the NPDS. If scales have different numbers of categories (steps), a strong parallel reliability requires a high level of agreement in the ordering of all individuals involved. Agreement in the ordering of individuals between two scales' assessments is an important condition for scales to be interchangeable. To assess this, observed distribution is compared with the pattern of total agreement in the ordering of all individuals, since the rank ordering of all individuals in the RTPA is independent of the two scales [10]. A change from one scale to another then means a change in categorical labelling but does not mean an alteration in the relative ordering of the individuals. The RTPA is completely defined by the two sets of marginal distributions and is constructed by pairing off the two sets of marginal distribution.

## 3. Results

The sensitivity of the NPDS as compared to FIM varied between 0.53 (feeding) and 1.0 (mobility) and specificity, between 0.64 (mobility) and 1.0 (bladder). The positive predictive value for NPDS compared to FIM varied from 0.62 (mobility) to 1.0 (bladder) and the negative predictive value varied from 0.48 (bowel) to 1.0 (mobility) (Table 1). Agreement between the scales with kappa analyses was moderate to good (four items) and excellent (three items) (Table 2).

**Figure 1:**
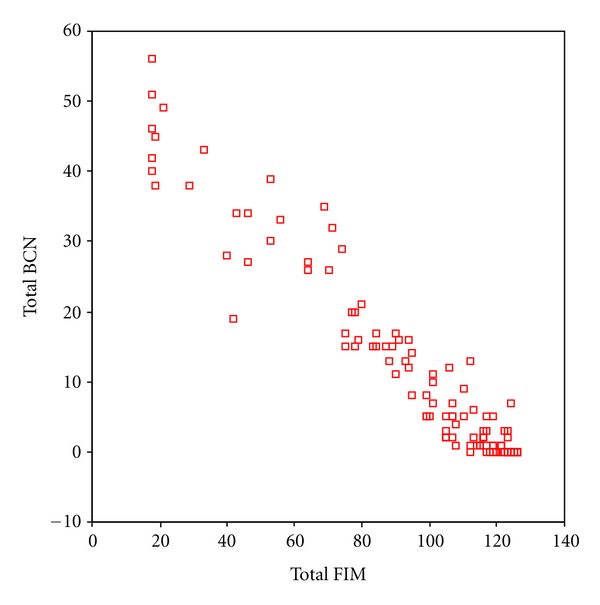
Scatterplot showing the distribution of the NPDS total BCN score and the FIM total score.

Concordance was good, with a gamma of −.856, an asymptotic error (ase) of .025 and *P* < .000 (Figure 1). The parallel reliability of NPDS and FIM was tested with RTPA. The observed distribution (the total score in the BCN section of NPDS and the total score in FIM) was compared with the total agreement of the total score of each patient (Table 3), where the tendency was for the BCN section of NPDS to have more categories (steps) than FIM when the dependency is high (making it possible to differentiate between persons) and for the FIM to have more categories than NPDS when the dependency is low.

In terms of the total score of the BCN section of NPDS and the total score of FIM in the different items, concordance showed good agreement in all items, with the following distribution: item transfer gamma −.909 and ase .025. The self-care item had a gamma of −.867 and an ase of .030, the continence item a gamma of −.833 and an ase of .040, and the cognitive item a gamma of −.790 and an ase of .043.

Corresponding ICF domains could be found for all items in both instruments (Table 4). However, the instruments differed when the linking was performed to the fourth level of the ICF (Table 5). The NPDS-BCN section covers items in the sections on sensory functions and pain, which FIM does not. FIM covers items in the areas of learning and applying knowledge, which are not covered in NPDS.

## 4. Discussion

The key findings of the study are that the NPDS compared with the gold standard FIM had good instrumental qualities. Also NPDS is a good tool of the dependency among severely impaired patients when needs of nursing time are high.

In this work, the convergent validity of the NPDS to FIM was shown to be good. The different areas of basic ADL were good, with high sensitivity and specificity, and there was also good agreement. This confirms the conclusions drawn by Plantinga et al. [6], where a comparison was made with the Barthel index. In that study, however, only a portion of the sample was neurologically impaired (35% of 154), which is different from the present sample.

The diverse qualities of FIM and NPDS for differentiating patients' dependency were shown with the RTPA. One of findings is that the NPDS is more sensitive for change in the more severely disabled patients. However, if the patient is more independent, the FIM is more likely to detect further improvements. This means that, on a ward where the aim is to assess patients' needs, the two instruments are not interchangeable but are complementary since there is usually a variation in patients' functional limitations in any single ward.

Differences and similarities become clearer when two scales are linked to the ICF. This is a way to validate scales by content. The results of the linking give some clues as to why the scales behave differently, that is, why some patients' scores according to the different scales showed that they were more or less dependent. The linking shows that the contents of the items are not the same in spite of the fact that they aim to assess the same area.

One limitation in this study is the relative small sample. However, all patients suffered from neurological disorders, which make the sample representative in that respect. The sample also has a dominance of patients that function quite well and are not highly dependent in many areas. This reflects the selection of patients that receive in-patient rehabilitation in. It is possible that the results would be different in another rehabilitation setting.

## 5. Conclusion

The correlation between total FIM and the BCN section of the NPDS is high. FIM and NPDS may be used to complement one another. There are potential benefits of using NPDS as a measure of how sensitive the dependency is among severely injured patients when needs of nursing time are high. 

## Figures and Tables

**Table 1:** Sensitivity, specificity, positive predictive value, and negative predictive value of the NPDS, with FIM as the golden standard.

	Sensitivity	Specificity	Positive predictive value	Negative predictive value
Feeding	0.53	0.93	0.94	0.69
Toileting	0.83	0.98	0.98	0.87
Dressing	0.89	0.98	0.98	0.88
Bladder	0.72	1.0	1.0	0.55
Bowel	0.74	0.88	0.96	0.48
Mobility	1.0	0.64	0.62	1.0
Showering	0.83	0.98	0.97	0.87

**Table 2:** Agreement between the FIM and the NPDS for different basic ADL areas.

	Kappa value	Ase	Sign
Feeding	0.653	0.068	0.000
Toileting	0.803	0.057	0.000
Dressing	0.860	0.047	0.000
Bladder	0.566	0.071	0.000
Bowel	0.457	0.080	0.000
Mobility	0.570	0.067	0.000
Showering	0.824	0.053	0.000

**Table 3:** The rank-transformable pattern of agreement (RTPA) of the total score of FIM and the BCN section of NPDS.

FIM	NPDS
18	46
29	38
40	35
46	32–28
56–64	27
69	21
75–77	20
78	19–17
79-80	17
84–87	16
88–90	15
91	14
93-94	13
95	11-12
99	8
100-101	7
105-106	5
107	3–5
108-109	3
112–115	2
116–121	1
122–126	0

**Table 4:** Commonality of items in the FIM and the NPDS.

FIM	NPDS
FIM motor	Basic care needs
*Self-care *	*Feeding *
Eating	Eating
	Drinking
	Enteral feeding
	*Washing*, *bathing*, *dressing *
Grooming	Washing and grooming
Bathing	
Bathing/showering	
Dressing upper body	Dressing
Dressing lower body	
Toileting	
* Continence *	* Continence *
Bladder	Toileting-bladder
Bowel	Urinary incontinence
	Toileting-bowels
	Faecal incontinence
	
*Transfers *	*Mobility and transfers *
Bed, chair, wheelchair	Mobility
Toilet	Transfers
Tub, shower	
*Locomotion *	
Walk, wheelchair	
Stairs	
FIM social/cognitive	*Safety*, *communication*, *behavior *
	Skin pressure relief
	Safety awareness
*Communication *	Communication
Comprehension	
Expression	
*Social cognition *	
Social interaction	Behavior
Problem solving	
Memory	Special nursing needs
	Tracheotomy
	Open wound requiring dressing
	Requires 2 interventions at night
	Requires psychological support
	In isolation (e.g., for MRSA screening)
	Acute medical/surgical intervention
	Needs one-to-one “specializing”

**Table 5:** Comparing FIM and NPDS to the International Classification of Functioning, Disability, and Health.

ICF	FIM	ICF	NPDS-BCN section
	Self-care	d 560, 445, 440	Drinking
d 550, 560	Eating	d 550	Eating
		b 510	Enteral feeding
d 510, 520	Grooming	d 510, 520	Washing and grooming
d 510	Bathing	d 510, 450, 465	Bathing/showering
d 540	Dressing upper body	d 540	Dressing
d 540	Dressing lower body		
d 530	Toilet	d 530	Toilet bladder
		d 530	Toilet bowels
	Sphincter control		
b 620	Bladder management	b 620	Urinary incontinence
b 525	Bowel management	b 525	Faecal incontinence
	Transfer		Bed transfer
d 420, 410	Bed, chair, wheelchair	d 450, 410, 415	
d 420, 410	Toilet	d 420	requires hoisting
d 420	Tub, shower		
	Locomotion		Mobility
d 450, 455, 465	Walk, wheelchair	d 450	Walk
d 455	Stairs	d 465	With equipment
		b 270	Skin pressure relief
	Communication	d 310, 315	Communication
d 310, 315	Comprehension	d 335	Gestures, contextual cues
d 330, 335	Expression		
	Social, cognition		
d 710	Social interaction	d 720, b 130	Behaviour
d 230, 175	Problem solving	b 114	Safety awareness
b 144	Memory		
			Special nursing needs—SNN
		b 265, 270, 280	
		b 810	

b: Body function.

d: Activity performance.
